# Item Response Patterns on the Patient Health Questionnaire-8 in a Nationally Representative Sample of US Adults

**DOI:** 10.3389/fpsyt.2017.00251

**Published:** 2017-11-24

**Authors:** Shinichiro Tomitaka, Yohei Kawasaki, Kazuki Ide, Maiko Akutagawa, Hiroshi Yamada, Ono Yutaka, Toshiaki A. Furukawa

**Affiliations:** ^1^Department of Mental Health, Panasonic Health Center, Tokyo, Japan; ^2^Department of Health Promotion and Human Behavior, Kyoto University Graduate School of Medicine/School of Public Health, Kyoto, Japan; ^3^Department of Drug Evaluation and Informatics, School of Pharmaceutical Sciences, University of Shizuoka, Shizuoka, Japan; ^4^Clinical Research Center, Chiba University Hospital, Chiba, Japan; ^5^Department of Pharmacoepidemiology, Graduate School of Medicine and Public Health, Kyoto University, Kyoto, Japan; ^6^Center for the Promotion of Interdisciplinary Education and Research, Kyoto University, Kyoto, Japan; ^7^Center for the Development of Cognitive Behavior Therapy Training, Tokyo, Japan

**Keywords:** exploratory data analysis, depressive symptoms, Patient Health Questionnaire-8, item response, behavioral risk factor surveillance survey, exponential distribution, end-digit preference bias

## Abstract

Recent studies have shown that item responses on the Center for Epidemiologic Studies Depression Scale (CES-D) and Kessler Screening Scale for Psychological Distress (K6) exhibit the same characteristic item response patterns among the general population. However, the distributional patterns of responses on the Patient Health Questionnaire-8 (PHQ-8) among the general population have not been adequately studied. Thus, we conducted a pattern analysis of PHQ-8 item responses among US adults. Data (18,446 individuals) were obtained from the 2015 Behavioral Risk Factor Surveillance Survey (BRFSS). Item responses on the BRFSS version of the PHQ-8 were scored using the number of days response set and then converted to the original 4-point scale. The patterns of item responses were analyzed through graphical analysis. Lines of item responses scored using the number of days response set showed the same pattern among the eight items, characterized by crossing at a single point between “0 days” and “1 day,” and parallel fluctuation from “1 day” to “14 days” on a semi-logarithmic scale. Lines of item responses converted to the 4-point scale also showed the same characteristic pattern among the eight items. The present results demonstrate that the item responses on the PHQ-8 show the same characteristic patterns among items, consistent with the CES-D and the K6.

## Introduction

Major depression is a common but serious psychological disorder that affects more than 300 million people around the world (1, 2). Moreover, major depression is frequently associated with suicidal behavior (3). Since the diagnosis of major depression is based on the severity of depressive symptoms, the severity distribution of depressive symptoms in a general population has been of great interest to researchers (4).

Epidemiological studies of depressive symptoms have been conducted intensively using a variety of depression screening scales (5–7). These studies have provided important information on depressive symptoms in the general population (e.g., the estimated prevalence of clinical depression). However, little has been reported regarding the distributional patterns of item responses on depression screening scales among the general population. Information about the mathematical patterns of item responses on depression screening scales is important for several reasons. First, it predicts how depressive symptoms distribute among the general population. If the mathematical pattern of item responses adequately approximates the empirical distribution, the pattern is useful for evaluating the scores of individuals in a population and verifying the survey results. Second, the mathematical pattern determines which statistical methods to use (e.g., parametric or non-parametric statistics) (8). Generally, parametric statistics, which assume a normally distributed latent variable, are widely used to analyze psychological scale data. However, there has been no reliable evidence to date that the item responses to depression screening scales follow a normally distributed latent variable (9). If the empirical distributions of item responses follow a non-normal distribution, the statistical model of normal variables will require reconsideration. Finally, if the distributions of depressive symptoms are found to follow a specific pattern, it will provide further insight into the mechanism of depressive symptomatology.

**Figure 1:**
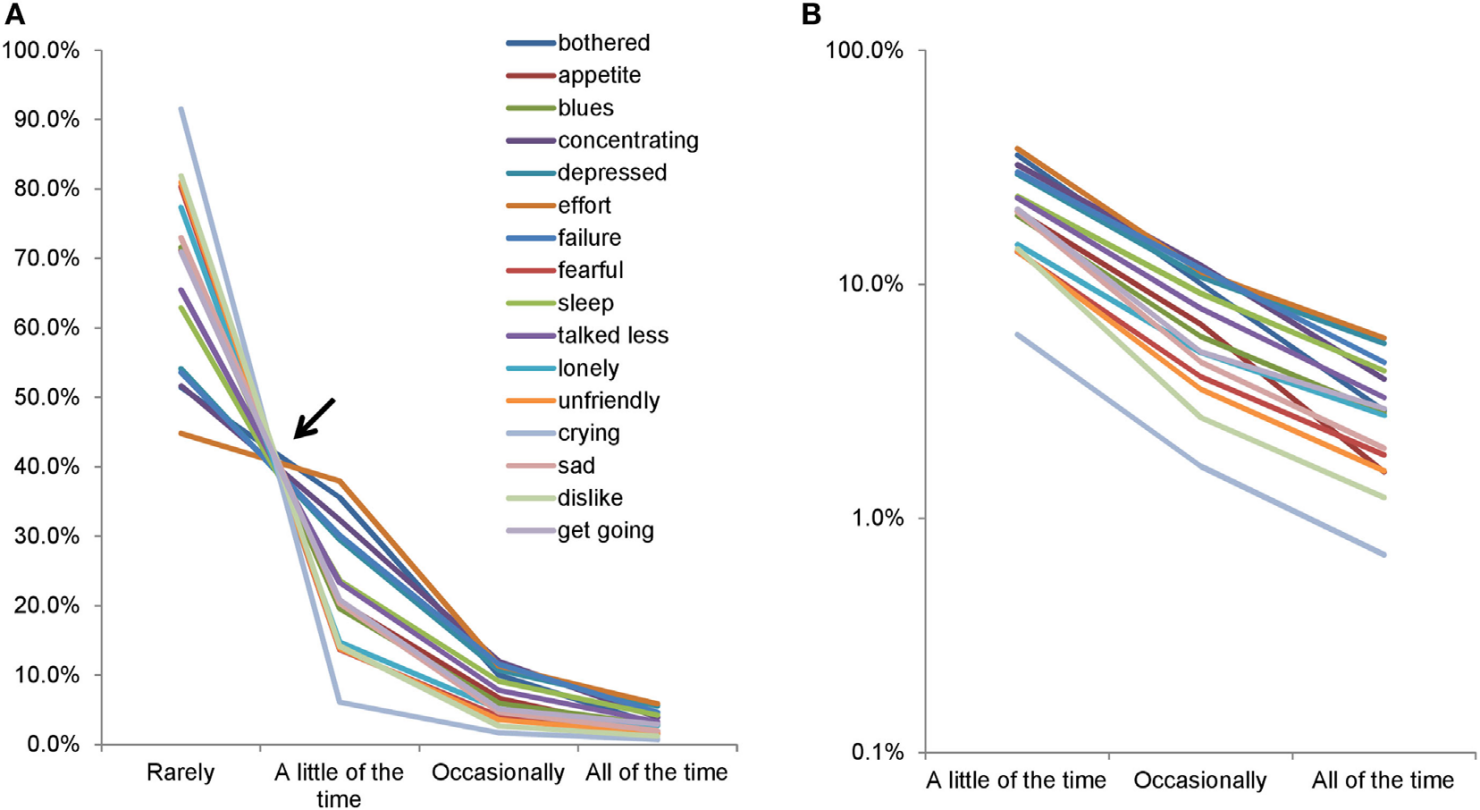
Item responses of the Center for Epidemiologic Studies Depression Scale. Item responses for the 16 items are presented using a normal scale **(A)** and a semi-logarithmic scale **(B)**. **(A)** The lines for the 16 items crossed between “rarely” and “a little of the time.” Between “a little of the time” and “all of the time,” the same lines showed a decreasing pattern. **(B)** The lines for the 16 items exhibited a parallel linear pattern between “a little of the time” and “all of the time” on a semi-logarithmic scale. Image credit: PLoS ONE, https://doi.org/10.1371/journal.pone.0165928.g001.

In general, large sample sizes allow researchers to better identify the mathematical pattern of a sample distribution. Analyzing data from about 32,000 respondents in a national survey of the Japanese population, we first observed that responses to the Center for Epidemiologic Studies Depression Scale (CES-D) exhibited a common mathematical pattern among the 16 depressive symptom items (Figure 1) (10, 11). The CES-D measures the frequency of each depressive symptom over the past seven days with response options of “rarely,” “a little of the time,” “occasionally,” and “all of the time” (12). As shown in Figure 1, the lines for the 16 items cross at a single point between “rarely” and “a little of time,” after which they exhibit a decreasing pattern (Figure 1A). Using a semi-logarithmic scale, the lines of the item responses decrease in parallel from “some” to “most of the time” (Figure 1B) (10). Moreover, another study using CES-D data from 8,000 Japanese employees further replicated the common mathematical pattern of item responses observed in the previous study (13). Mathematically, if the ratios of “occasionally” to “a little of the time,” and “all of the time” to “occasionally,” are the same among all items, the lines for item responses can cross at a single point between “rarely” and “a little of the time,” and exhibit a parallel pattern from “some” to “all of the time” on a semi-logarithmic scale. In fact, the ratios of “occasionally” to “a little of the time,” and “all of the time” to “occasionally,” were similar among all items in the previous studies (10, 11).

To confirm the reproducibility of such findings for another depression screening scale, we investigated the item responses on the Kessler Screening Scale for Psychological Distress (K6) in representative US studies. Although the K6 is a broad measure of psychological distress, the K6 has been used as a screening tool for depression (14). The K6 comprises six items that ask about the frequency with which participants felt, during the past 30 days, sad, nervous, restless, hopeless, that everything was an effort, and worthless. Each item is rated on a 5-point scale with the options of 0 = none of the time, 1 = a little of the time, 2 = some of the time, 3 = most of the time, and 4 = all of the time. In an analysis of a large data sample from the National Survey of Midlife Development in the United States (MIDUS), we consistently found that the lines for the six items between “none of the time” and “a little of the time” crossed at a single point, after which they decreased in parallel from “a little of the time” to “all of the time” on a semi-logarithmic scale (15). Taken together, these results suggest that item responses on the CES-D and K6 exhibit the same characteristic patterns across all items.

The Patient Health Questionnaire-9 (PHQ-9) is one of the most commonly used measures for depression screening worldwide (16, 17). The PHQ-9 measures the nine criteria for depression from the *Diagnostic and Statistical Manual of Mental Disorders*, Fifth Edition (DSM-5) (18). The Patient Health Questionnaire-8 (PHQ-8) was developed for the 2006 Behavioral Risk Factor Surveillance Survey (BRFSS) (19). The PHQ-8 omits the ninth item of the PHQ-9 regarding passive thoughts of death and active ideas of self-harm, because interviewers may not be able to provide adequate intervention. The PHQ-8 data from the BRFSS are suitable for the reproducibility test of the aforementioned findings because of the large sample sizes and limited selection bias. BRFSS data are freely available to researchers worldwide and have been used in hundreds of papers in public health (20).

The original PHQ-8 allows individuals to self-rate the frequency of various depressive symptoms over the past 2 weeks using a 4-point verbal scale: “not at all,” “several days,” “more than half the days,” and “nearly every day.” However, on the BRFSS version of the PHQ-8, respondents were asked to self-rate the number of days of each depressive symptom during the past 14 days; then, this number was converted to the original 4-point scale of the PHQ-8 (19). Thus, we can determine the distributions of item responses using the number of days response set, potentially enabling a further understanding of the relationship between the pattern of item responses and response sets. To detect the pattern of item responses, we visualized the pattern with histograms. Visualizations are central to exploratory data analysis because the rich information they provide is unrivaled in its ability to reveal data patterns (21).

For the present study, we elucidated the characteristics of item responses on the PHQ-8 using the number of days response set and determined whether they exhibited similar patterns across all items. After confirming that item responses using the number of days response set followed the characteristic pattern, we analyzed the pattern of item responses converted to the original 4-point response set.

## Materials and Methods

### Data Set

Data were obtained from the 2015 BRFSS (22). The BRFSS is a population-based state surveillance system that uses random-digit-dialed telephone surveys of noninstitutionalized US adults. The BRFSS gathers data on the behaviors and conditions that put people at risk for chronic disease. State health departments operate the BRFSS in partnership with the Centers for Disease Control and Prevention. The BRFSS completes more than 400,000 adult interviews each year, making it the largest continuously conducted health survey system in the world. As noted in the Section “Introduction,” BRFSS data are freely available to researchers worldwide (20).

The BRFSS questionnaire comprises three parts: (1) a standard set of questions asked in all 50 states and the District of Columbia, consisting of queries about health-related perceptions, conditions, and behaviors; (2) optional modules, which are sets of questions on specific topics (e.g., anxiety and depression, excess sun exposure, cancer survivorship); and (3) state-added questions. The demographic variables of the BRFSS questionnaire include age, sex, education, marital status, employment, income, and race/ethnicity. All BRFSS data are available on the website (22).

In 2015, four states conducted the optional Anxiety and Depression Module (ADM): Mississippi, North Dakota, Tennessee, and West Virginia. Therefore, the analyses in this study are limited to data from those four states. The ADM includes the PHQ-8. The total 2015 ADM sample comprised 22,943 respondents, including 6,035, 4,972, 5,979, and 5,957 respondents for Mississippi, North Dakota, Tennessee, and West Virginia, respectively. The response rates for Mississippi, North Dakota, Tennessee, and West Virginia were 49.9, 58.9, 38.6, and 48.9%, respectively. Detailed descriptions of the sociodemographic characteristics of the BRFSS respondents are reported elsewhere (22).

### Ethics Statement

Our institutional review board does not consider the analysis of publicly available data as research involving human subjects. Since this study used a de-identified, publicly available data set, institutional review board approval was not required.

### Measures

As noted in the Section “Introduction,” the PHQ-8 reflects eight of the nine criteria on which DSM-5 diagnosis of major depressive disorder is based. The PHQ-8 response set in the BRFSS was standardized to other BRFSS questions by using the number of days of each depressive symptom during the past 14 days. The number of days response set was converted to the original PHQ-8 response set: 0–1 day = not at all; 2–6 days = several days; 7–11 days = more than half the days; and 12–14 days = nearly every day. Points were assigned to each category (0–3) (19).

### Analysis

First, we analyzed the distributions of item responses using the number of days response set. The pattern of item responses for the eight items was visualized with histograms (normal scales and a semi-logarithmic scale). Mathematically, if the ratios between two consecutive response options are the same among all items, all lines for the item responses will exhibit a parallel pattern on a semi-logarithmic scale (10). Since the ratios between two consecutive response options, except for the lower end option, were similar among all items in previous studies (10, 15), all ratios between two consecutive response options from 1 to 14 days were calculated for all eight items.

After confirming that the item responses using the number of days response set followed the same pattern among the eight items, consistent with previous studies, we analyzed the distributions of item responses converted to the original 4-point response set. Analyses were conducted using JMP version 11 for Windows (SAS Institute, Inc., Cary, NC, USA).

## Results

### Demographic Characteristics of the Participants

Of the 22,943 respondents, those who did not report the number of days during the past 14 days for all eight items (*n* = 4,497) were excluded from the analysis. The final sample comprised 18,446 respondents (7,514 men) with an average age of 56.1 years old [ages 18–29: *n* = 1,639 (male, *n* = 798); ages 30–39: *n* = 1,864 (male, *n* = 809); ages 40–49: *n* = 2,267 (male, *n* = 979); ages 50–59: *n* = 3,338 (male; *n* = 1,800); ages 60–69: *n* = 4,592 (male, *n* = 1,578); ages 70–79: *n* = 2,820 (male, *n* = 1,060); and age 80 or older: *n* = 1,426 (male, *n* = 444)].

### Item Responses Using the Number of Days Response Set

Table 1 depicts item response rates using the number of days response set for all eight items. The highest frequency was observed for 0 days for all eight items. From 1 to 13 days, there was a general decreasing tendency with the increase in the number of days. However, increased frequencies were observed at 14 days for all eight items.

**Table 1:** Item responses scored by the number of days response set.

Item	Number of days, %														
	0	1	2	3	4	5	6	7	8	9	10	11	12	13	14
Loss of interest	70.0	4.3	7.0	3.2	2.2	2.2	0.7	2.2	0.5	0.2	1.7	0.0	0.4	0.1	5.2
Feeling depressed	75.1	4.6	5.6	2.6	1.5	1.8	0.4	1.9	0.5	0.1	1.2	0.0	0.3	0.0	4.4
Sleep problems	54.4	3.9	7.0	4.7	3.4	3.1	1.3	2.9	0.9	0.2	2.4	0.1	0.7	0.2	15.0
Loss of energy	40.1	6.1	12.1	6.5	4.3	4.5	1.4	3.7	1.0	0.2	2.9	0.1	0.7	0.2	16.2
Appetite problems	65.4	4.2	6.5	4.1	2.5	2.8	0.9	2.4	0.5	0.1	1.7	0.0	0.3	0.1	8.5
Self-blame	83.3	2.9	3.2	1.5	0.9	1.1	0.3	1.0	0.4	0.0	0.8	0.0	0.2	0.0	4.4
Concentration problems	85.6	1.5	2.8	1.3	1.1	1.1	0.3	1.2	0.2	0.1	0.6	0.0	0.2	0.0	3.8
Agitation/retardation	89.1	1.2	1.9	1.3	0.8	0.9	0.3	0.9	0.2	0.1	0.5	0.0	0.1	0.0	2.8

**Figure 2:**
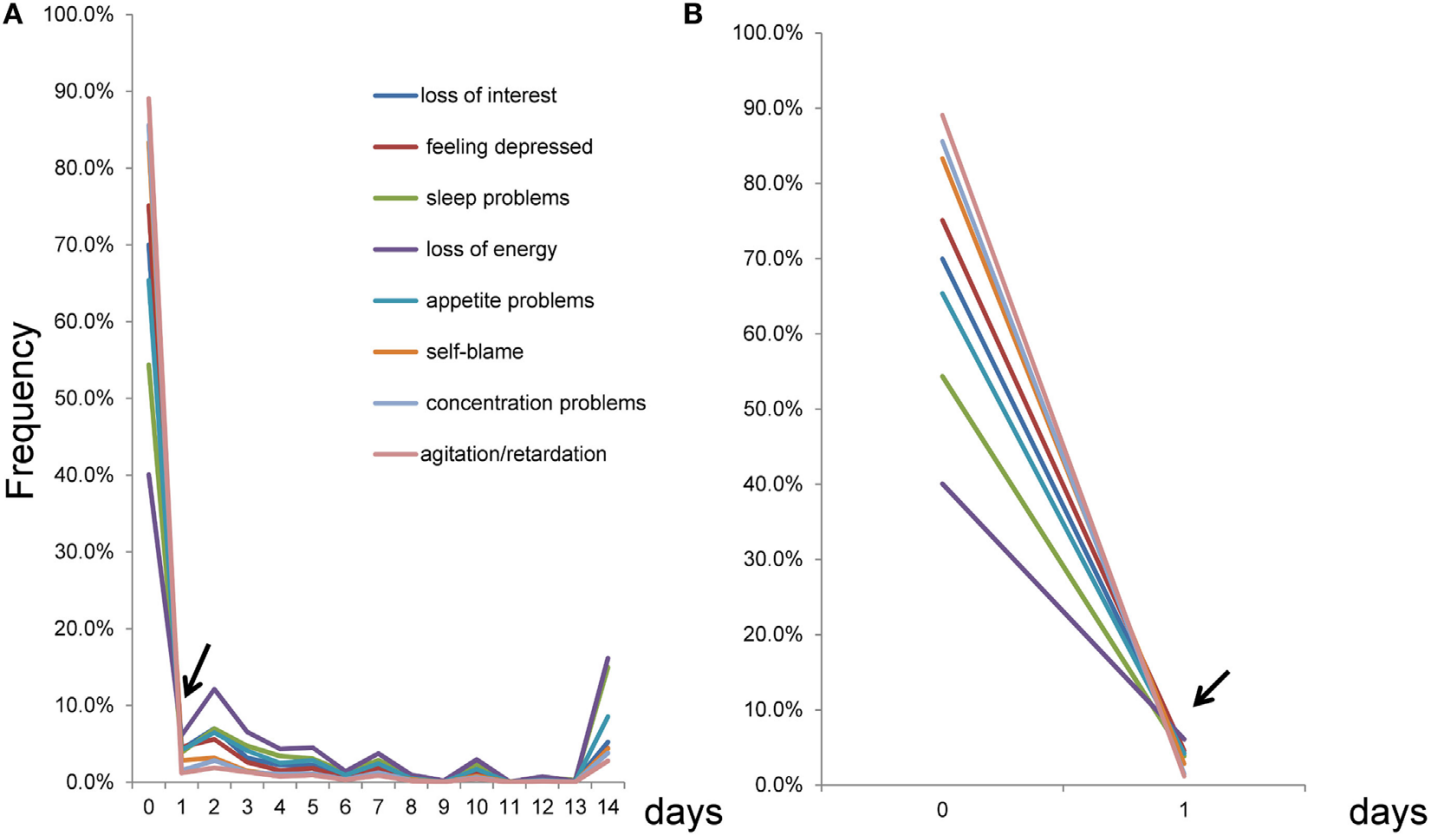
Item responses scored using the number of days response set from 0 to 14 days. **(A)** As indicated by the arrow, the lines of the eight items crossed at a single point near 1 day, while the lines seemed to fluctuate in synchrony from 1 to 14 days. **(B)** An enlarged version of **(A)** between 0 and 1 day. As indicated by the arrow, the lines for the eight items crossed at a single point between 0 and 1 day.

To assess the pattern of item responses using the number of days, all eight item response frequencies were plotted on the same scale (Figure 2). As indicated by the arrow, lines of the eight items crossed at a single point near “1 day” (Figure 2A). Conversely, lines for the eight items fluctuated in synchrony from 1 to 14 days. To examine the crossing point carefully, the section of Figure 2A between 0 and 1 day was enlarged. It was evident that the lines of item responses crossed at a single point between 0 and 1 day (Figure 2B), consistent with Figure 1A.

**Figure 3:**
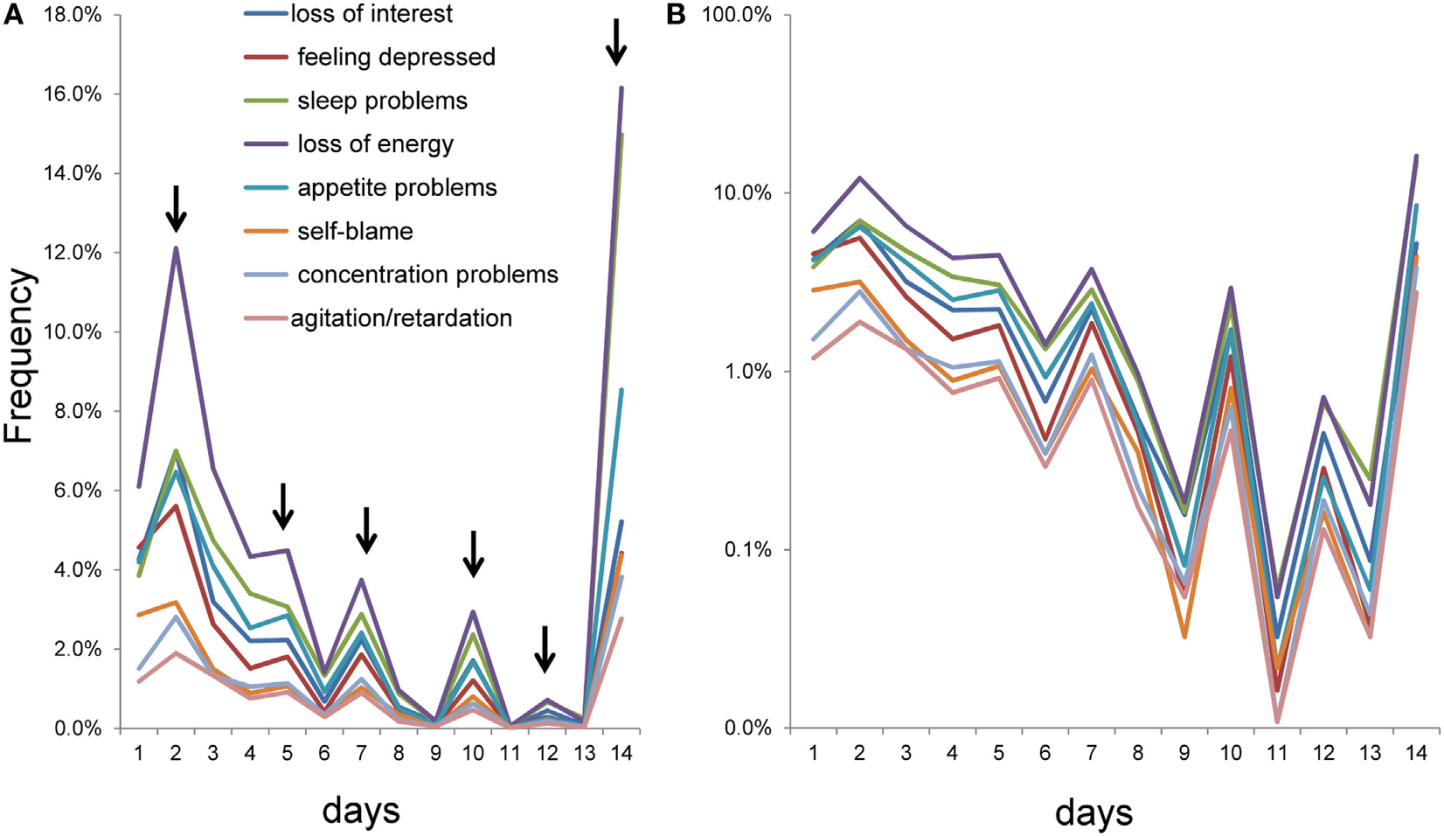
Item responses scored using the number of days response set from 1 to 14 days. **(A)** As the arrows indicate, the eight items showed peaks at 2, 5, 7, 10, 12, and 14 days, while valleys were observed at 6, 9, 11, and 13 days. **(B)** Using a semi-logarithmic scale, the lines of item responses showed parallel fluctuation from 1 to 14 days.

To further examine the patterns of item responses for the eight items, a graph was constructed for 1–14 days (Figure 3). The item responses of the eight items exhibited similar patterns of peaks and valleys for 1–14 days. As the arrows indicate, the eight items showed peaks at 2, 5, 7, 10, 12, and 14 days. In contrast, valleys were observed at 6, 9, 11, and 13 days (Figure 3A).

Using a semi-logarithmic scale, lines of the item responses showed parallel fluctuation from 1 to 14 days (Figure 3B). As noted in the Section “Introduction,” the parallelism of the eight lines on a semi-logarithmic scale represents the similarity of the ratios between two consecutive response options among the eight items. In fact, all ratios between two consecutive response options from 1 to 14 days were similar to some extent among all items (Table 2). Although the item responses from 0 to 14 days were complex, the graphical analysis revealed that item responses exhibited a common pattern among the eight items (Figure 3).

**Table 2:** Ratios of frequencies between adjacent numbers of days.

Item	Rate of 2–1 day	Rate of 3–2 days	Rate of 4–3 days	Rate of 5–4 days	Rate of 6–5 days	Rate of 7–6 days	Rate of 8–7 days	Rate of 9–8 days	Rate of 10–9 days	Rate of 11–10 days	Rate of 12–11 days	Rate of 13–12 days	Rate of 14–13 days
Loss of interest	1.6	0.5	0.7	1.0	0.3	3.3	0.2	0.3	10.9	0.02	13.8	0.2	60.1
Feeling depressed	1.2	0.5	0.6	1.2	0.2	4.5	0.2	0.1	22.4	0.01	17.7	0.1	116.6
Sleep problems	1.8	0.7	0.7	0.9	0.4	2.2	0.3	0.2	14.6	0.03	11.3	0.4	60.1
Loss of energy	2.0	0.5	0.7	1.0	0.3	2.6	0.3	0.2	15.9	0.02	13.2	0.3	90.3
Appetite problems	1.5	0.6	0.6	1.1	0.3	2.6	0.2	0.2	20.7	0.01	11.8	0.2	143.2
Self-blame	1.1	0.5	0.6	1.2	0.3	3.0	0.3	0.1	24.8	0.03	7.5	0.2	134.5
Concentration problems	1.9	0.5	0.8	1.1	0.3	3.6	0.2	0.3	9.7	0.02	17.5	0.2	88.0
Agitation/retardation	1.6	0.7	0.6	1.2	0.3	3.1	0.2	0.3	8.6	0.02	12.0	0.3	85.0
Average	1.6	0.6	0.7	1.1	0.3	2.8	0.3	0.2	14.9	0.02	12.6	0.3	83.6
SD	0.3	0.1	0.1	0.1	0.1	0.7	0.1	0.1	6.1	0.01	3.3	0.1	31.4

### Item Responses Converted to the 4-Point Response Set

Table 3 shows the item response rates converted to the 4-point response set. A common tendency was observed for all eight items, with the highest frequency being for “not at all,” followed by a decreasing frequency with the increase in item scores, and finally an increased frequency again for “nearly every day.” The ratios of “more than half the days” to “several days,” and “nearly every day” to “more than half the days,” were similar to some extent among the eight items.

**Table 3:** Item responses converted to the 4-point response set.

Item	Item response, %				Rate of “more than half the days” to “several days”	Rate of “nearly every day” to “more than half the days”
	Not at all (0–1 days)	Several days (2–6 days)	More than half the days (7–11 days)	Nearly every day (12–14 days)		
Loss of interest	74.2	15.3	4.7	5.8	0.31	1.23
Feeling depressed	79.7	12.0	3.6	4.7	0.30	1.32
Sleep problems	58.2	19.5	6.4	15.9	0.33	2.50
Loss of energy	46.2	28.9	7.9	17.0	0.27	2.16
Appetite problems	69.6	16.9	4.7	8.9	0.28	1.88
Self-blame	86.2	7.0	2.2	4.6	0.32	2.03
Concentration problems	87.1	6.7	2.2	4.0	0.32	1.87
Agitation/retardation	90.3	5.2	1.6	2.9	0.31	1.82
Average	73.9	13.9	4.2	8.0	0.30 ± 0.02	1.85 ± 0.42

*Average rate data are presented as the mean ± SD*.

**Figure 4:**
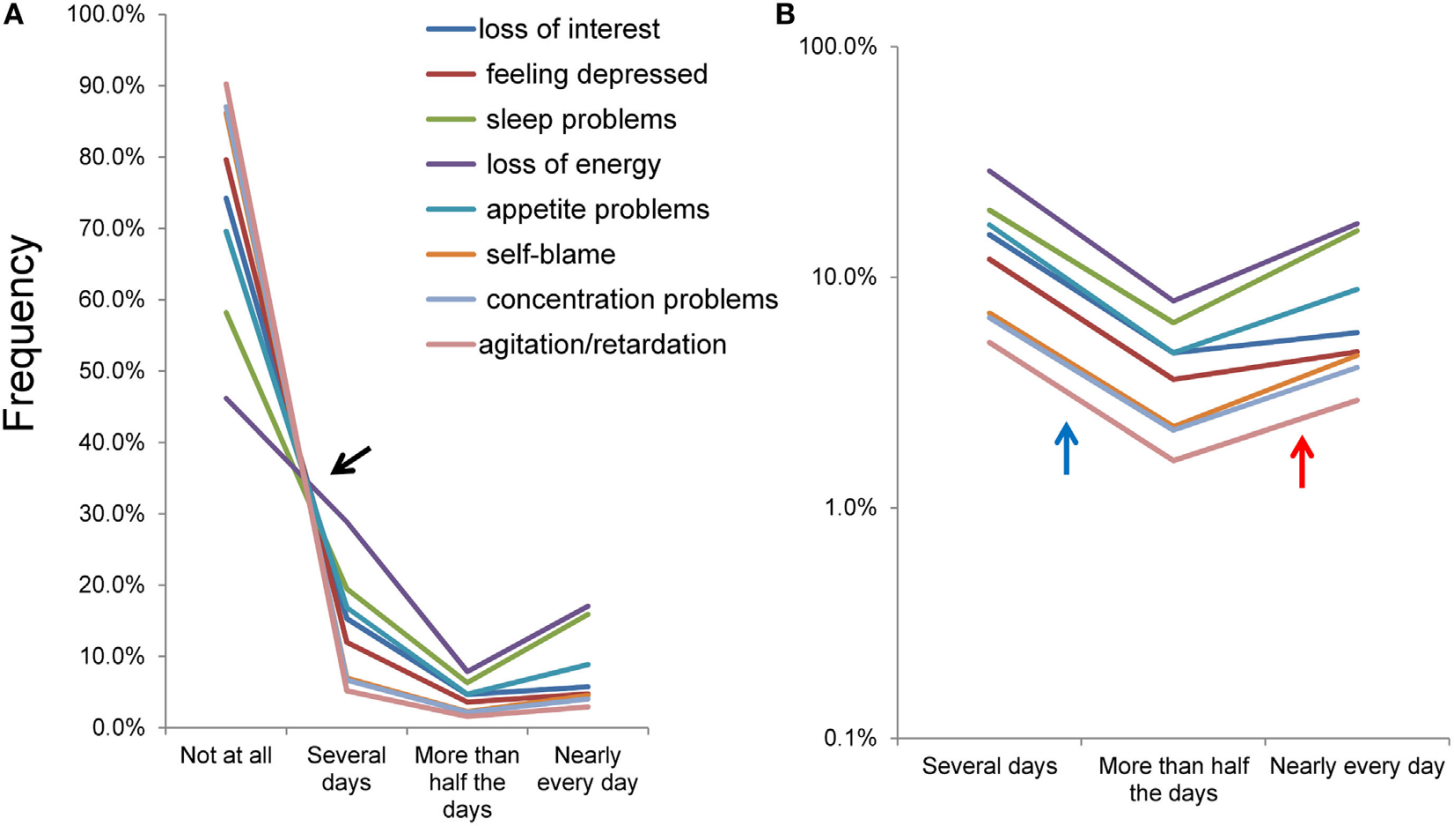
Item responses converted to the original 4-point response set. Item responses for the eight items of depressive symptoms exhibited a common mathematical pattern among the eight items on a normal scale **(A)** and a semi-logarithmic scale **(B)**. **(A)** Between “not at all” and “several days,” the lines for the eight items crossed in the vicinity of a single point. Conversely, between “several days” and “nearly every day,” the lines for the eight items seemed to barely cross and showed a V-shaped pattern. **(B)** Using a logarithmic scale, lines of the item responses showed a parallel V-shaped pattern from “several days” to “nearly every day.”

To identify the patterns of item responses, all eight item response rates were plotted on the same scale (Figure 4). The eight items exhibited a common pattern that shows different types of distributions with a boundary at “several days” (Figure 4A). Between “not at all” and “several days,” the lines for the eight items crossed in the vicinity of a single point, although the line for “loss of energy” was slightly away from the single point. Conversely, between “several days” and “nearly every day,” lines for the eight items seemed to barely cross and showed a V-shaped pattern.

Using a semi-logarithmic scale, the lines of the item responses showed a parallel V-shaped pattern from “several days” to “nearly every day” (Figure 4B). The gradient of the linear pattern of item responses moved downward between “several days” and “more than half the days” (blue arrow), and upward between “more than half the days” and “nearly every day” (red arrow). These observations are in line with the average ratio of “more than half the days” to “several days” (0.30) being smaller compared to that of “nearly every day” to “more than half the days” (1.85).

## Discussion

This study’s main finding is that the item responses on the PHQ-8 using the number of days response set showed a common pattern among the eight items. The pattern was characterized by the lines crossing at a single point between “0 days” and “1 day,” and parallel fluctuation from “1 day” to “14 days,” on a semi-logarithmic scale. Although the pattern from “1 day” to “14 days” on the semi-logarithmic scale was complicated, the fluctuation pattern appeared to be a series of parallel V-shaped patterns. This is consistent with the item responses using the 4-point response set—as well as the results of other studies using the CES-D and K6 (10, 15)—in that the lines for the item responses cross at a single point between the option at the lower end and the adjacent option, and show a parallel pattern for the remaining options on a semi-logarithmic scale.

As noted in the Section “Introduction,” the common pattern of the item responses on depression screening scales can be explained mathematically. If all ratios between two consecutive response options, except for the lower end, are the same among all items, the lines for item responses will cross at a single point between the option at the lower end and the adjacent option, and show a parallel pattern for the remaining options on a semi-logarithmic scale (10). Of note, since lognormal scales evaluate the frequencies of item responses after logarithmic transformation, small differences in the ratios between two consecutive days do not have a great effect on the parallelism of the eight lines. Thus, although the ratios between two consecutive days from 1 to 14 days were slightly different among the eight items (Table 2), we could observe parallel fluctuations from “1 day” to “14 days” on a semi-logarithmic scale (Figure 3B).

Item responses using the number of days response set showed a similar pattern of peaks and valleys among the eight items. Peaks at 5, 7, 10, and 12 days, and adjacent valleys, suggest that some of the peaks and valleys were subject to end-digit preference bias (23). Interestingly, despite the influence of end-digit preference, the parallelism of the eight lines was preserved from 1 to 14 days. The peaks and valleys suggest that the “distance” between each successive response option (number of days) is not equivalent. In view of the theory that the latent trait of depressive symptom items follows an exponential distribution (10), if the “distance” between each successive response option is equivalent, the response options (number of days) will have a geometric distribution.

Although the PHQ-8, CES-D, and K6 differ in terms of item content and response set (12, 14, 24), the item responses of the three scales exhibit different patterns between the response option at the lower end and the remaining options (11, 15). These different patterns can be discussed in terms of the threshold of each item. In general, each item on depression screening scales is self-rated in two stages. First, each participant assesses whether the given symptoms are present. If the severity of each symptom does not meet the threshold at which the participant notices, then it is categorized as “absence” (i.e., it occurs “not at all” on the PHQ-8). Second, if the depressive symptom meets the threshold, its duration is categorized according to the remaining degree-adverb options (i.e., it occurs “several days,” “more than half the days,” or “nearly every day”). This two-step process suggests that “absence” will cover the under-threshold range while the remaining degree-adverb options cover the fixed proportion of the above-threshold range (10). If the latent variable of depressive symptoms follows an exponential distribution, and each of the remaining response options cover the fixed proportion of the above-threshold range, each response rate of depressive symptoms may show the mathematical pattern observed in this study. However, further research is needed to clarify how each of the remaining response options covers the fixed proportion of the above-threshold range.

In line with previous studies, the present results do not indicate that the latent variable of depressive symptom scores follows a normal distribution. However, normality-assuming statistics (e.g., Pearson correlation coefficient) are widely used in population studies of depressive symptoms (25, 26). To our knowledge, there is no evidence that the latent variable of depressive symptom scores in the general population follows a normal distribution. Given that the pattern of item responses has repeatedly shown a non-normal distribution, the normal distribution model of depressive symptom scores could require reconsideration (27).

This research has some limitations. First, while we investigated the pattern of item responses using graphical analysis, we could not quantify the degree of similarity of item responses. Since the pattern of item responses was complicated (especially item responses using the number of days response set), it was difficult to apply unitary regression analysis to the item responses. In general, the quantification of similarity is more difficult with more complex patterns. In the case of a simple and unitary pattern, we can use existing distribution models (normal, linear, exponential, etc.) and easily calculate the goodness of fit using unitary regression analysis. Conversely, since the pattern of item responses is a collection of a number of sub-patterns, we must assess the similarities in all parts of the sub-patterns and consider the results of the similarities of all parts together. To our knowledge, there is no standard statistical procedure that integrates the results of the similarities of all parts in a complex pattern. In short, we think quantifying the similarities of the complex patterns in this study is an issue for future research.

Next, the PHQ-8 omits the ninth item of the PHQ-9. It is unclear whether the finding that the item responses on depression screening scales follow a common pattern can be generalized to suicide-related items. Further studies employing assessment tools such as the Suicide History Self-Rating Screening Scale are needed to determine whether suicide-related items follow the same item response patterns as other items (3).

However, our research has methodological advantages. First, although the methods of this study were simple (visualizations using histograms), they allowed us to observe a complex pattern of item responses; this could have been unsuccessful if the item responses had not been visualized with histograms. Generally speaking, graphical analysis is crucial for exploratory data analysis involving complex patterns (28, 29). Next, as noted in the Introduction, using BRFSS data with a large sample size enabled us to identify the pattern of distribution. Since all data were available to the researchers, they could easily review the present findings using the raw data.

Finally, we come to the significance and potential application of our study. First, with regard to descriptive statistics, if the mathematical patterns for item responses on the PHQ-8 are established, the distributions of item responses can be described using mathematical models. Consequently, we can easily evaluate the patterns of item responses with estimated parameters. Previous epidemiological studies have paid little attention to the patterns of item responses in depression screening scales, partly because there were no models to describe the patterns of item responses. Since researchers understand the characteristics of data through models, the use of mathematical models will enable us to observe further findings (30). In fact, in the present study, we found that item responses using the number of days response set were subject to the same end-digit preference bias among the eight items. Without knowledge of the mathematical patterns, we would not have been able to observe these findings.

Second, regarding inference statistics, the distributional patterns of item responses are significant because statistical hypothesis tests and statistical estimators are derived from statistical models, which are assumed to adequately approximate the empirical distribution. As noted in the Introduction, if the empirical distributions of item responses follow a non-normal distribution, the statistical model of normal variables will require reconsideration.

Finally, evidence that the mathematical patterns of item responses on the PHQ-8, CES-D, and K6 are the same will provide further insight into the mechanism of depressive symptoms. Given the same mathematical pattern among all depressive symptoms, we assumed that all depressive symptom items shared one latent trait. In clinical settings, depressive symptoms are assessed using depression rating scales and added to construct sum scores. These scores are used as a proxy for depression severity. To allow for such interpretations, depression rating scales must measure a single construct (31). Although prior studies using factor analysis are highly inconsistent regarding the question of unidimensionality in depression rating scales (31, 32), our research supports a unidimensional latent trait.

## Author Contributions

ST carried out the design of the study and the statistical analysis and wrote the manuscript. YK, KI, MA, and HY contributed to the analysis of the data. OY and TF contributed to the acquisition of data. YK, KI, MA, HY, OY, and TF interpreted the data and wrote the manuscript. All authors read and approved the final manuscript.

## Conflict of Interest Statement

The authors declare that the research was conducted in the absence of any commercial or financial relationships that could be construed as a potential conflict of interest.
